# Sun Tracking Systems: A Review

**DOI:** 10.3390/s90503875

**Published:** 2009-05-20

**Authors:** Chia-Yen Lee, Po-Cheng Chou, Che-Ming Chiang, Chiu-Feng Lin

**Affiliations:** 1 Department of Materials Engineering, National Pingtung University of Science and Technology, Pingtung, Taiwan 912; E-Mail: leecy@mail.npust.edu.tw; 2 Department of Interior Design, Shu-Te University, Kaohisung County, Taiwan 824; E-Mail: paul@mail.stu.edu.tw; 3 Department of Architecture, National Cheng Kung University, Taiwan 700; E-Mail: cmchiang@mail.ncku.edu.tw; 4 Department of Vehicle Engineering, National Pingtung University of Science and Technology, Pingtung, Taiwan 912

**Keywords:** solar orientation, solar position, sun sensor algorithm, sun tracking system

## Abstract

The output power produced by high-concentration solar thermal and photovoltaic systems is directly related to the amount of solar energy acquired by the system, and it is therefore necessary to track the sun's position with a high degree of accuracy. Many systems have been proposed to facilitate this task over the past 20 years. Accordingly, this paper commences by providing a high level overview of the sun tracking system field and then describes some of the more significant proposals for closed-loop and open-loop types of sun tracking systems.

## 1. Introduction

Solar energy systems have emerged as a viable source of renewable energy over the past two or three decades, and are now widely used for a variety of industrial and domestic applications. Such systems are based on a solar collector, designed to collect the sun's energy and to convert it into either electrical power or thermal energy. The literature contains many studies regarding the use of solar collectors to implement such applications as light fixtures, window covering systems, cookers, and so forth [1-6]. In general, the power developed in such applications depends fundamentally upon the amount of solar energy captured by the collector, and thus the problem of developing tracking schemes capable of following the trajectory of the sun throughout the course of the day on a year-round basis has received significant coverage in the literature. For example, various schemes have been proposed for optimizing the tilt angle and orientation of solar collectors designed for different geographical latitudes or possible utilization periods [7-8]. In general, the results showed that by using mathematical models to optimize the tilt angle and orientation of the solar collector, a yearly gain of more than 5% could be obtained in the captured solar radiation compared to the case in which the collector was fixed on a horizontal surface. In Aden city (Yemen), the improvement in the performance of a solar cooker during summer was found to be as much as 40% for higher elevation angle and 70% for lower elevation angle, based on the developed tracking algorithms in [5]. Moreover, it was shown in [9] that the amount of solar energy captured by a tilted collector could be increased by more than 40% by adjusting the tilt angle on a seasonal basis.

In 1975, one of the first automatic solar tracking systems [10-13] was presented by McFee, in which an algorithm was developed to compute total received power and flux density distribution in a central receiver solar power system [10]. By subdividing each mirror into 484 elements and summing the contributions of all elements, the sun position could be determined with a tracking error tolerance of 0.5^°^ – 1^°^. Several years later, Semma and Imamru used a simple microprocessor to adaptively adjust the positions of the solar collectors in a photovoltaic concentrator such that they pointed toward the sun at all times [13]. Mathematical theories of tracking error distributions were also developed to improve the algorithms of determining sun position [14,15].

With rapid advances in the computer technology and systems control fields in recent decades, the literature now contains many sophisticated sun tracking systems designed to maximize the efficiency of solar thermal and photovoltaic systems. Broadly speaking, these systems can be classified as either closed-loop or open-loop types, depending on their mode of signal operation (Table 1). The remainder of this paper presents a systematic review of the operational principles and advantages of each of the major closed-loop and open-loop types of sun tracking systems presented in the literature over the past 20 years.

## 2. Closed-loop Types of Sun Tracking Systems

**Figure 1.**
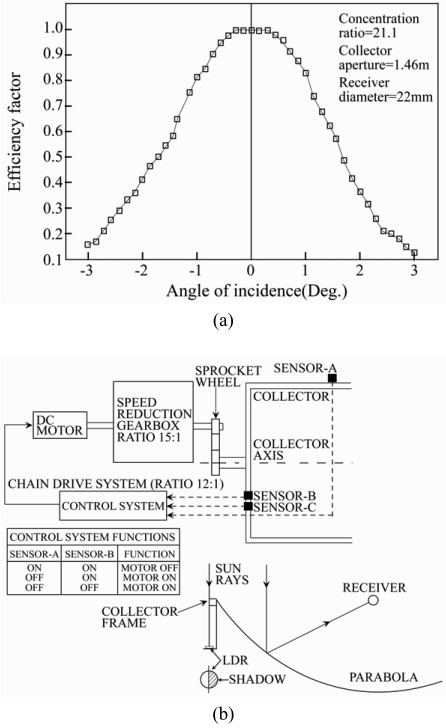
(a) Collector acceptance angle. (b) illustration of sun tracking mechanism. Reproduced with permission from Elsevier [21].

Closed-loop types of sun tracking systems are based on feedback control principles. In these systems, a number of inputs are transferred to a controller from sensors which detect relevant parameters induced by the sun, manipulated in the controller and then yield outputs (i.e. sensor-based). In 1986, Akhmedyarov *et al.* [16] first increased the output power of a solar photoelectric station in Kazakhstan from 357 W to 500 W by integrating the station with an automatic sun tracking system. Several years later, Maish [17] developed a control system called SolarTrak to provide sun tracking, night and emergency storage, communication, and manual drive control functions for one- and two-axis solar trackers in a low-cost, user-friendly package. The control algorithm used a six-degree self-alignment routine and a self-adjusting motor actuation time in order to improve both the pointing accuracy and the system reliability. The experimental results showed that the control system enabled a full-day pointing accuracy of better than ±0.1^°^ to be achieved. In 1992, Agarwal [18] presented a two-axis tracking system consisting of worm gear drives and four bar-type kinematic linkages to facilitate the accurate focusing of the reflectors in a solar concentrator system. In the same year, Enslin [19] applied the principles of maximum power point tracking (MPPT) to realize a power electronic converter for transforming the output voltage of a solar panel to the required DC battery bus voltage. An MPPT system consists of two basic components: a switchmode converter and a control/tracking section. The switchmode converter is the core of the entire system and allows energy at one potential to be drawn, stored as magnetic energy in an inductor, and then released at a different potential. By setting up the switchmode section in various different topologies, either high-to-low or low-to-high voltage converters can be constructed. The goal of an MPPT system is to provide a fixed input voltage and/or current, such that the solar panel is held at the maximum power point, while allowing the output to match the battery voltage. In [19], the converter was controlled to track the maximum power point of the input source under varying input and output parameters and was shown to provide a minimum input source saving of 15% for 3-5 kWh/day systems. Brown and Stone [20] developed a tracking system for solar concentrators in which a neural network was applied to an error model in order to compensate for tracking errors. The test data showed that the resulting system was capable of reducing the tracking error to a value of less than 0.01^°^ (0.2 mrad). Kalogirou [21] presented a one-axis sun-tracking system utilizing three light-dependent resistors (LDRs). The first LDR detected the focus state of the collector, while the second and third LDRs were designed to establish the presence (or absence) of cloud cover and to discriminate between day and night, respectively. The output signals from the three LDRs were fed to an electronic control system which actuated a low-speed 12 - V DC motor in such a way as to rotate the collector such that it remained pointed toward the sun (Figure 1). In 1997, Stone and Sutherland [22] presented a multiple tracking measurement system comprising more than 100 heliostats for tracking the sun's position on an hourly basis from early morning to late evening. Hua and Shen [23] compared the solar tracking efficiencies of various MPPT algorithms and implemented a simple control method which combined a discrete time control scheme and a proportional-integral (PI) controller to track the maximum power points (MPPs) of a solar array.

**Figure 2.**
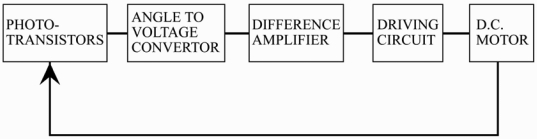
Block diagram of sun-tracking system. Reproduced with permission from Elsevier [24].

In 1998, Khalifa and Al-Mutawalli [24] developed a two-axis sun tracking system to enhance the thermal performance of a compound parabolic concentrator. The system was designed to track the sun's position every three to four minutes in the horizontal plane and every four to five minutes in the vertical plane. As shown in Figure 2, the tracking system was comprised of two identical sub-systems, one for each axis, with each sub-system consisting of two adjacent photo-transistors separated by a partition of a certain height. In the tracking operation, the difference in the voltage signals of the two photo-transistors was amplified and used as a command signal to drive the collector around the corresponding axis until the voltage difference reduced to zero, indicating that the sun's rays were once again normal to the collector surface. It was shown that the tracking system had a power consumption of just 0.5 Whr and yielded an improvement of around 75% in the collected solar energy, compared to a fixed collector of equivalent dimensions. Yousef [25] developed a sun tracking system in which the nonlinear dynamics of the tracking mechanism were controlled using a fuzzy logic control algorithm implemented on a PC and supported by an interfacing card consisting of a sensor data acquisition function, motor driving circuits, signal conditioning circuits and serial communications. Kim *et al.* [26] proposed an enhanced incremental conductance (IncCond) MPPT control algorithm for determining the maximum power operation point (MPOP) of a photovoltaic power system subject to rapidly changing levels of solar radiation. It was shown that the decision regarding the MPOP could be rendered robust to short-term fluctuations in the photocurrent by inserting a test signal in the control input. Falbel *et al.* [27] presented a sun-oriented attitude-control system combined with a concentrating solar panel for use in a satellite (CUBESAT). The solar sensor had the form of a two-axis analog device, which measured the sun's location relative to its optical axis based on the differential signal obtained from a quadrant silicon detector upon which a circular spot generated by the sun's irradiance was imaged. The calibration results showed that the sensor was capable of locating the position of the sun with an accuracy of ±0.05^°^. Urbano *et al.* [28] presented a 5 Watt-PV module for a stand-alone solar tracking system with a capacity of 2.6 kW. The tracking system was designed to follow the position of the sun autonomously in the altitude and azimuth directions and was driven by two 12 V DC motors, each with a power consumption of 36 W and both fed by a single electrolytic condenser charged by the PV module. Jiang and Cao [29] constructed an emulated sunflower based on a spherical four-quadrant photoelectric sensor for solar tracking purposes. The sunflower was designed in such a way that when the sun's rays were aligned with the normal direction of the detector surface, the photocurrents produced by the rays incident in each quadrant were equal to one another. However, any changes in the sun's position produced a differential change in the output signals of each quadrant. It was shown that through an appropriate manipulation of the four output signals, a control signal could be produced to drive the position of the detector such that the difference between the output signals was once again restored to zero. Luque-Heredia *et al.* [30] presented a sub-degree precision sun tracker for 1,000X micro-concentrator modules. The tracking system comprised a lightweight structure designed to remain operative for high wind speeds, yielded 95% of the available direct solar radiation and featured an electronic tracking control unit which relied on an adaptive algorithm for absorbing unforeseen or time varying errors with automatic calibration of cheap sun pointing sensors against array power output.

**Figure 3.**
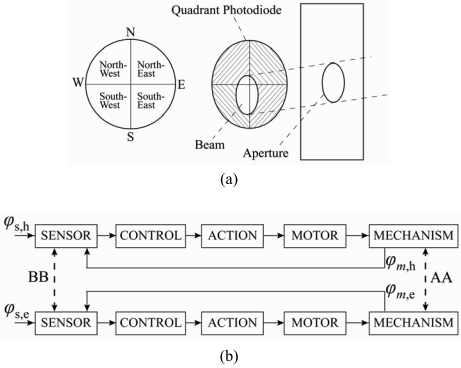
(a) four-quadrant sensor and (b) transfer functions for both axes. Reproduced with permission from Elsevier [31].

**Figure 4.**
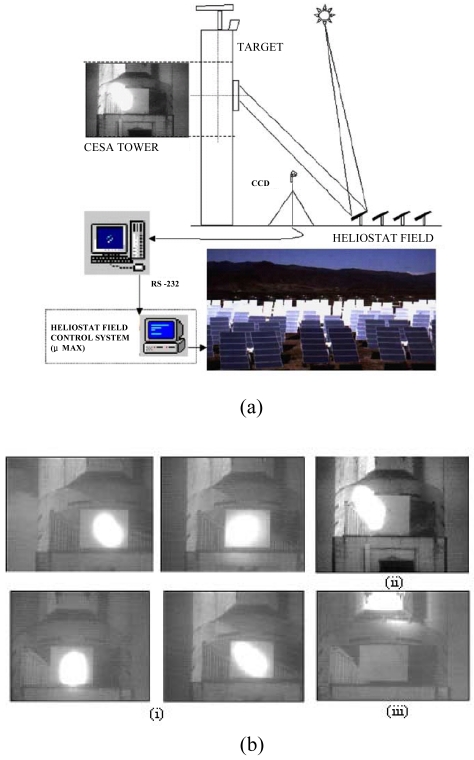
(a) Schematic illustration of vision-based heliostat control system, and (b) photographs showing different shapes of sun images projected by heliostats onto target plane: (i) centered ellipsoids (the shape of the ellipsoid changes during the day), and (ii), (iii) ellipsoids outside of target boundaries due to aiming errors. Reproduced with permission from Elsevier [33].

In 2004, Roth *et al.* [31, 32] designed and constructed a sun tracking system in which a pyrheliometer was used to measure the direct solar radiation. The system was controlled by a closed-loop servo system consisting of a four-quadrant photodetector (Figure 3(a)) to sense the sun's position and two small DC motors to drive the instrument platform in such a way that the sun's image remained at the center of the four-quadrant detector at all times. Note that the cross-coupling of AA and BB in Figure 3(b) is virtually zero due to the orthogonal disposition of the axes and the parallel mounting of the sensor. In the same year, Berenguel *et al.* [33] developed an automatic heliostat offset correction control system based on an artificial vision technique and common charge-coupled device (CCD) equipment (Figure 4(a)). In the proposed approach, a B/W CCD camera captured images of the sun projected from the heliostats with a resolution of 640 × 380 or 768 × 576 (Figure 4(b)) and supplied the images in real time to a computer via a frame-grabber with a PCI bus. The images were then compared with reference images in which the sun's rays were incident in a normal direction to the heliostat surface. The difference between the two images was used to compute a command signal, which was then passed to the heliostat control system and used to actuate the heliostat servomotors in such a way that the heliostat surfaces were restored to an angle of 90 degrees to the sun's rays.

Abdallah [34] investigated the respective effects of four different electro-mechanical sun-tracking systems on the current, voltage and power characteristics of a flat-plate photovoltaic system. The results showed that tracking systems comprising two axes, one vertical axis, one east-west axis and one north-south axis, and one north-south axis, increased the electrical output powers of the photovoltaic system by around 43.87%, 37.53%, 34.43% and 15.69%, respectively, compared to that obtained from a photovoltaic system with a fixed surface inclined at 32^°^ to the north. Al-Mohamad [35] used a programmable logic controller (PLC) to control a photovoltaic module for following the sun's radiation. It was shown that by collecting and storing the data relating to the sun's radiation, and using this information to control the photovoltaic module, the daily output power of the photovoltaic system could be improved by more than 20% relative to that obtained from a fixed module.

**Figure 5.**
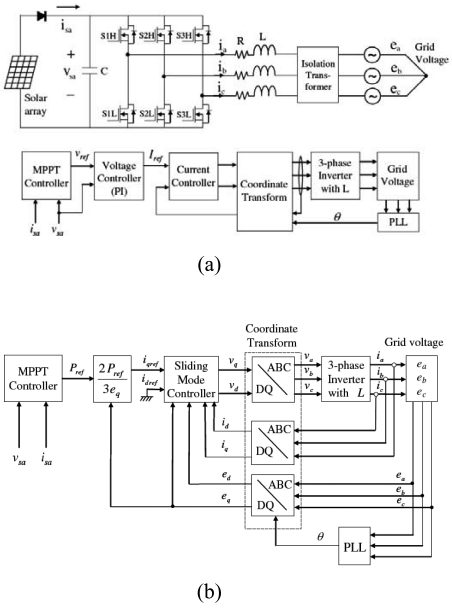
(a) Typical configuration of three-phase grid-connected PV system, (b) overall configuration of system controller. Reproduced with permission from Elsevier [38].

Aiuchi *et al.* [36] presented a simple sun tracking photo-sensor designed to ensure a constant direction of the reflected solar radiation. In the proposed device, two photo-cells were placed side by side at the bottom of a box with an aperture. When the reflected solar radiation passed through the aperture, the photo-cells were fractionally illuminated and produced an electric current proportional to the size of the illuminated area. A constant direction of the reflected solar radiation was maintained simply by monitoring the output signals of the two photo-cells and adjusting the angle of the reflection mirror as required to ensure that the two signals remained equal at all times. It was shown that the resulting system achieved a tracking error of less than 0.6 mrad on a sunny day. In 2005, Alata *et al.* [37] designed and simulated three sun tracking systems, namely: (1) one-axis sun tracking with the tilted aperture equal to the latitude angle, (2) equatorial two-axis sun tracking, and (3) azimuth/elevation sun tracking. For each tracking system, the modeling and controller design tasks were accomplished using the first-order Sugeno fuzzy inference system. In addition, the insolation incident on the two-axis sun tracking system was determined in accordance with fuzzy IF- THEN rules. Having generated the input/output data, a subtractive clustering algorithm and a Least Square Estimation (LSE) scheme were applied to generate a set of fuzzy rules with which to predict the solar angles given the local time. Finally, the fuzzy rules were tuned by an Adaptive Neuro-Fuzzy Inference System (ANFIS) and implemented in an open-loop control system. In 2007, Kim [38] presented a robust MPPT system based on a sliding mode controller (SMC) for a three-phase grid-connected photovoltaic system. The proposed system comprised both a MPPT controller and a current controller (Figure 5). The MPPT controller generated a current reference directly from the solar array power information, while the current controller used an integral sliding mode scheme to ensure a tight control of the current. The proposed system demonstrated a robust tracking performance in the presence of both modeling uncertainties and parameter variations.

## 3. Open-loop Types of Sun Tracking Systems

An open-loop type of controller computes its input into a system using only the current state and the algorithm of the system and without using feedback to determine if its input has achieved the desired goal (i.e. algorithm-based). The system is simpler and cheaper than the closed-loop type of sun tracking systems. It does not observe the output of the processes that it is controlling. Consequently, an open-loop system can not correct any errors so that it could make and may not compensate for disturbances in the system. Open-loop control algorithms of sun tracking systems utilize some form of solar irradiation geometry model [39].

In 1983, Al-Naima and Yaghobian [40] developed a solar tracking system featuring a two-axis equatorial mount and a microprocessor, in which the tracking operation was performed on the basis of the astronomical coordinates of the sun. The experimental results demonstrated that the proposed system yielded a significantly better tracking performance than that obtained by a conventional sensor- controlled system. Several years later, Lorenz [41] proposed a set of design guidelines for a window glazing which rejected solar radiation during the summer, but accepted it during the winter. The design featured a purely passive control algorithm based on seasonal changes in the incident angle of the solar rays.

Blanco-Muriel *et al.* [42] argued that sun-tracking systems in which open-loop controllers are used to compute the direction of the solar vector should be both highly accurate (in order to enhance the solar concentration efficiency) and computationally straightforward (to minimize the price of the tracking system). Having reviewed existing solar vector prediction algorithms, the authors developed a new algorithm for predicting the solar vector given a knowledge of the time (given as the date and the Universal Time) and the location (given as the longitude and latitude of the observer in degrees). The performance of the proposed algorithm was verified by evaluating the direction of the sun vector for 447,048 reference values of the true horizontal coordinates of the sun over the period 1999∼2015. It was shown that the algorithm enabled the direction of the solar vector to be determined with an error of less than 0.5 minutes of arc. Table 2 compares the sun vectors generated by the proposed algorithm with those computed using the algorithm proposed by Michalsky in 1988 [43]. Overall, the results show that the estimates obtained from the proposed algorithm for the azimuth and zenith angle of the sun are approximately 15% and 22%, respectively, better than those obtained from the algorithm presented in [43]. In 2003, Beshears *et al.* [44] presented a micro controller-based sun positioning system for hybrid lighting applications in which the celestial bearing of the sun with respect to the earth was computed directly from a knowledge of the local time, date, latitude, longitude and time zone information.

**Figure 6.**
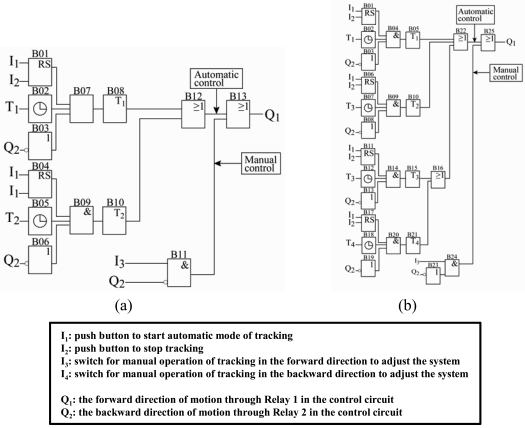
Functional PLC program for plane rotated about (a) south-north axis and (b) about vertical axis. Reproduced with permission from Elsevier [45].

In 2004, Abdallah and Nijmeh [45] developed an electro-mechanical, two-axis tracking system in which the motion of the sun tracking surface was controlled by an open-loop control algorithm implemented using a PLC unit. The proposed system incorporated two separate tracking motors, namely one motor to rotate the sun tracking surface about the horizontal north-south axis, i.e. to adjust the slope of the surface (Figure 6(a)) and the other to rotate the sun tracking surface about the vertical axis, i.e. to adjust the azimuth angle of the surface (Figure 6(b)). The experimental results indicated that the two-axis tracking system increased the total daily energy collection by approximately 41.34% compared with that obtained from a fixed surface tilted at 32^°^ towards the south.

**Figure 7.**
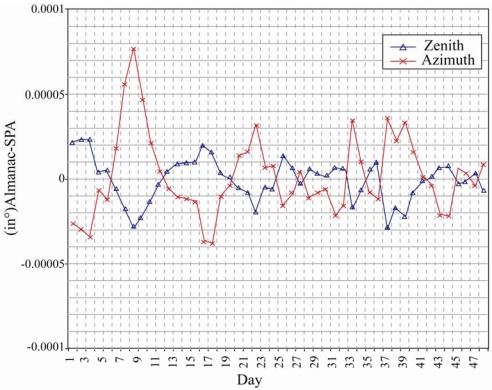
Uncertainties in solar zenith and azimuth angles. Reproduced with permission from Elsevier [46].

In the same year, Reda and Andreas [46] presented a simple step-by-step procedure for implementing a solar position algorithm. In the proposed algorithm, the solar zenith, azimuth and incidence angles were derived using the following main parameters: ecliptic longitude and latitude for mean Equinox of date, apparent right ascension and apparent declination, together with the following correction parameters: nutation in longitude, nutation in obliquity, obliquity of ecliptic and true geometric distance. The results showed that the solar zenith and azimuth angles could be calculated with uncertainties of ±0.0003^°^ (Figure 7).

**Figure 8.**
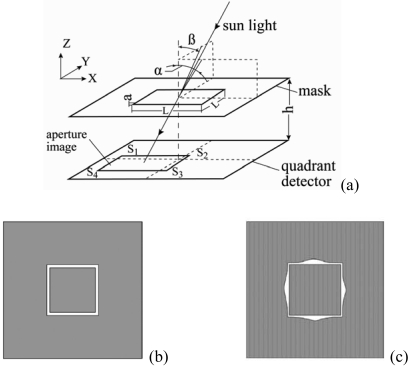
(a) Operational principle of traditional analogue sun sensor, (b) aperture of traditional analogue sun sensor, (c) aperture of proposed analogue sun sensor. Reproduced with permission from IOP Publishing Ltd. [48].

In 2007, Chen *et al.* [47,48] presented a sun sensor algorithm based on an analogue optical nonlinear compensation measuring principle. In a traditional analogue sun sensor (Figure 8(a)), a thin mask with a square aperture consisting of four slits of equal width (Figure 8(b)) is placed above a quadrant detector. The incident sunlight illuminates different positions of the detector depending on its angle relative to the main sensor axis and forms a projective image on the detector's plane. The signal generated by each quadrant, i.e. S_1_, S_2_, S_3_ and S_4_ (Figure 8(a)), is directly proportional to the illuminated area within that quadrant. Thus, in the traditional analogue sun sensor, the azimuth and elevation angles of the sun, i.e. α and β, respectively, are derived by a signal processing scheme in accordance with basic geometrical principles, i.e.:

(1) $$\alpha {=\tan}^{-1}(\frac{L}{h},M),\;\left|M\right|\leq \frac{L-a}{2L}$$

(2) $$\beta {=\tan}^{-1}(\frac{L}{h},N),\;\left|N\right|\leq \frac{L-a}{2L}$$

where $M=\frac{S_{1}+S_{4}-(S_{2}+S_{3})}{S_{1}+S_{2}+S_{3}+S_{4}}$, $N=\frac{S_{3}+S_{4}-(S_{1}+S_{2})}{S_{1}+S_{2}+S_{3}+S_{4}}$, *L* is the average length of the square aperture, *h* is the distance between the mask and the detector plane, and *a* is the width of the slit. However, in Eqs. (1) and (2), the output signals *M* and *N* vary nonlinearly with the inputs α and β, respectively, i.e. the sensitivity of the sensor depends upon the incident angle of the sunlight. To resolve this problem, the authors replaced the conventional aperture with that shown in Figure 8(c), in which the aperture area per unit length varied in accordance with specific laws. Thus, the nonlinear displacement of the projective image on the detector's plane caused by linear changes in the incident angle of the sunlight was compensated by the nonlinear aperture area per unit length such that the output of the sensor varied linearly with the input. The experimental results showed that the proposed sensor had an accuracy of better than 0.2^°^ over the entire field of view of ± 62^°^ for both axes.

**Figure 9.**
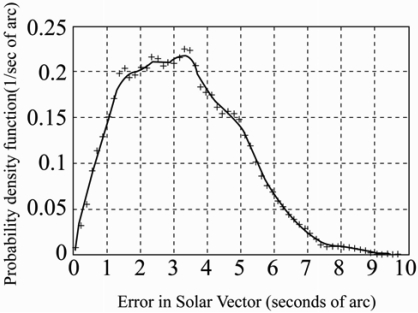
Distribution of errors in solar vector (i.e. solar vector: angular position of the sun). Reproduced with permission from Elsevier [49].

In a recent study, Grena [49] presented an algorithm for obtaining highly precise values of the solar position. Taking the fractional Universal Time (UT), the date, and the difference between UT and Terrestrial Time (TT) as inputs, the algorithm computed the angular position of the earth with respect to the sun in the ecliptic plane and then used this angle and the inclination angle of the earth's rotational axis to calculate the position of the sun. In the previous algorithms, the maximum error was ± 0.0003^°^ [46]. It was shown that the maximum error of the proposed algorithm, i.e. 0.0027^°^ (Figure 9), was higher than that of the algorithm presented by Reda and Andreas [46], i.e. ± 0.0003^°^, but was sufficient for most solar engineering applications and could be obtained at a fraction of the computational cost.

Recently, Chen *et al.* [50-51] and Chong *et al.* [52-53] presented general sun tracking formulas for open-loop type of sun tracking systems to solve the problem of any arbitrarily oriented sun-tracking axes for off-axis and on-axis solar collector respectively.

## 4. Conclusions

Advances in the algorithms of sun tracking systems have enabled the development of many solar thermal and photovoltaic systems for a diverse variety of applications in recent years. Compared to their traditional fixed-position counterparts, solar systems which track the changes in the sun's trajectory over the course of the day collect a far greater amount of solar energy, and therefore generate a significantly higher output power. This paper has presented a review of the major algorithms for sun tracking systems developed over the past 20 years. It has been shown that these sun tracking algorithms can be broadly classified as either closed-loop or open-loop types, depending on their mode of control. The control / computational principles of each method have been reviewed and their performance and relative advantages / disadvantages systematically discussed. Overall, the results presented in this review confirm the applicability of sun tracking system for a diverse range of high-performance solar-based applications.

## Figures and Tables

**Table 1.** Performance of sun tracking systems [16-53].

**Algorithm**	**References**	**Error**	**Gain in Energy Production Compared with a Non-tracking System**
Closed-loop Control	Akhmedyarov *et al.* (1986)	-	40%
	Maish (1990)	1^°^	-
	Enslin (1992)	-	10-15%
	Brown *et al.* (1993)	< 0.01^°^	-
	Kalogirou (1996)	0.05-0.2^°^	-
	Khalifa *et al.* (1998)	-	75%
	Falbel *et al.* (2002)	0.05^°^	-
	Al-Mohamad (2004)	-	20%
	Abdallah (2004)	-	15-44%
	Aiuchi *et al.* (2004)	0.1^°^	-

Open-loop Control	McFee (1975)	0.5-1^°^	-
	Blanco-Muriel *et al.* (2001)	0.08^°^	-
	Abdallah *et al.* (2004)	-	41%
	Reda *et al.* (2004)	0.0003^°^	-
	Chen F. *et al.* (2006)	0.02^°^	-
	Chen F. *et al.* (2007)	0.2^°^	-
	Grena (2008)	0.0027^°^	-
	Chong *et al.* (2009)	-	-

**Table 2.** Performance comparison of PSA and Michalsky algorithms used to predict sun's position over the period 1999-2015 [42,43].

	**Average**	**Standard Deviation**	**Mean Deviation**	**Range**

Error in Zenith Distance				
Michalsky	-0.128	0.137	0.109	[-0.666 0.340]
PSA Algorithm	-0.008	0.107	0.084	[-0.396 0.366]

Error in Azimuth				
Michalsky	-0.065	0.206	0.150	[-1.903 1.344]
PSA Algorithm	0.000	0.177	0.127	[-1.553 1.443]

Sun Vector Deviation				
Michalsky	0.208	0.110	0.086	[0.000 0.667]
PSA Algorithm	0.136	0.079	0.063	[0.000 0.433]
